# Does workplace health promotion contribute to job stress reduction? Three-year findings from *Partnering* Healthy@Work

**DOI:** 10.1186/s12889-015-2625-1

**Published:** 2015-12-24

**Authors:** Lisa Jarman, Angela Martin, Alison Venn, Petr Otahal, Kristy Sanderson

**Affiliations:** Menzies Institute for Medical Research, University of Tasmania, Private Bag 23, Hobart, 7000 TAS Australia; Tasmanian School of Business and Economics, University of Tasmania, Hobart, 7000 TAS Australia

**Keywords:** Stress, psychological, Workplace, Wellness programs, Health promotions

## Abstract

**Background:**

Workplace health promotion (WHP) has been proposed as a preventive intervention for job stress, possibly operating by promoting positive organizational culture or via programs promoting healthy lifestyles. The aim of this study was to investigate whether job stress changed over time in association with the availability of, and/or participation in a comprehensive WHP program (Healthy@Work).

**Method:**

This observational study was conducted in a diverse public sector organization (~28,000 employees). Using a repeated cross-sectional design with models corroborated using a cohort of repeat responders, self-report survey data were collected via a 40 % employee population random sample in 2010 (*N* = 3406) and 2013 (*N* = 3228). Outcomes assessed were effort and reward (self-esteem) components of the effort-reward imbalance (ERI) measure of job stress. Exposures were availability of, and participation in, comprehensive WHP. Linear mixed models and Poisson regression were used, with analyses stratified by sex and weighted for non-response.

**Results:**

Higher WHP availability was positively associated with higher perceived self-esteem among women. Women’s mean reward scores increased over time but were not statistically different (*p* > 0.05) after 3 years. For men, higher WHP participation was associated with lower perceived effort. Men’s mean ERI increased over time. Results were supported in the cohort group.

**Conclusions:**

For women, comprehensive WHP availability contributed to a sense of organizational support, potentially impacting the esteem component of reward. Men with higher WHP participation also benefitted but gains were modest over time and may have been hindered by other work environment factors.

**Electronic supplementary material:**

The online version of this article (doi:10.1186/s12889-015-2625-1) contains supplementary material, which is available to authorized users.

## Background

Job stress can lead to absenteeism [1] and presenteeism [2], and has been estimated to contribute to 40 % of all job turnover [3]. Evidence favours causal links between job stress and increased risk of down-stream illness [4–7]. The World Health Organisation cites workplace health promotion (WHP) as beneficial to job stress prevention, stating that health-promoting workplaces should address health at a systemic- (policies, practices, systems) as well as individual-level [8]. However, findings in favour of effective systems-level intervention to prevent job stress remain inconclusive [9, 10]. Evidence for stress prevention largely stems from individual-level stress management interventions [9–12].

Comprehensive WHP, a term given to interventions targeting both individual- and system-levels [13], has proven popular among employers, with associated decreases in absenteeism, presenteeism [14] and financial returns on investment [15, 16]. Nevertheless, publications citing research on comprehensive forms of WHP with job stress outcomes are uncommon [17] with the majority focusing on employee participation [18].

Conceptually, WHP appears associated with job stress in two key ways. First, investment in the ‘social capital’ of the organisation [19–21] may contribute to workers’ perceptions of support [19] from their organisation because the employer shows care for their health and well-being [22, 23]. The presence of WHP may also serve to reduce the stigma associated with reporting health-related issues or to enhance general health awareness among employees [23]. These emotional and cognitive effects have been linked to improved job satisfaction [18, 24] and mental health [25]. However, we were unable to identify any published articles that separated WHP availability from WHP participation when assessing whole-of-workforce job stress.

Second, exposure to job stress can provoke short-term behavioural responses such as inappropriate nutrition [26], smoking [27], physical inactivity [28] and alcohol consumption [29]. Extended exposure to situations stimulating stress responses can also lead to chronic arousal or strain [30]. Participation in workplace activities targeting known health risks or enhancing work-related coping strategies aims to reduce job stress. Meta-analytic research supports this link between participation in WHP programs, reduced job stress and improved mental health [9, 10, 18, 31]. Comprehensive strategies have also been shown to be more effective than approaches tackling only organisational or individual-level factors [32]. However, gaps remain in understanding time-related effects [17] and intervention effectiveness when WHP is scaled-up in size to intervene with whole working populations [33].

Effort-reward imbalance (ERI) concepts appear suited to assessing both pathways and a strong evidence-base is available across a broad range of occupations supporting the association between self-reported measures of ERI and enduring health outcomes such as cardiovascular disease [34, 35] and diabetes [36]. Effort-reward imbalance theory asserts that work is a form of mutual exchange, or reciprocity, where job-related efforts are traded for rewards (i.e., job security, career advancement, self-esteem) as a type of ‘social contract’. The theory proposes that insufficient reward for work effort can negatively impact upon the capacity of an individual to regulate their emotions, thoughts and behaviours, which in turn can lead to job strain [37]. Workplace health promotion may be viewed as an organisational benefit signaling regard for an employee’s welfare, thereby increasing perceived organisational support and enhancing self-esteem [19]. Research has highlighted that ERI measures explain unique variance in relation to the macro- or contractual factors contributing to mental health outcomes, and that the effort dimension can be likened to job-related demands [38, 39].

This project evolved from a collaboration between university researchers and government (public sector) that had the goal of evaluating the long-term effectiveness of a comprehensive multi-component WHP initiative, named *Healthy@Work*. Baseline workforce survey data had indicated that ERI was a key correlate of high psychological distress among employees, and that mental health varied by sex when compared with working population norms [40]. We hypothesized that i) higher availability of WHP would be positively associated with perceived reward, particularly through improved self-esteem (given job security and career progression were unlikely to be impacted by WHP), and ii) higher participation in WHP would be negatively associated with perceived effort.

## Methods

### Setting and study population

Tasmania is an Australian region with a population of around half a million people. The Tasmanian Government employed approximately 28,000 public sector workers across 14 separate organizations (government departments), which are highly diverse in their functions (e.g., health, education, fire services), locations (e.g., urban, rural, remote) and occupations. Participants were drawn from this working population and gave informed consent for study involvement, including publication. Ethics approval for the study was obtained from the Human Research Ethics Committee (Tasmania) Network (ID: H0010501).

### Intervention

#### Overview

Between 2009 and 2012, the Tasmanian Government invested approximately $2 million in a whole-of-workforce WHP intervention called Healthy@Work. A small, centralised Healthy@Work team was responsible for the associated structural changes including strategy, model development, principles and implementation cycle, and was tasked with oversight of this new government policy focus on WHP. Implementation was mandatory and was delegated to the senior executive of each government department. It was internally audited each year until its conclusion in mid-2012. Our research team commenced a partnership with the Tasmanian Government in 2010 to conduct a naturalistic evaluation of the intervention.

#### Department-based activities

Departments were responsible for establishing in-house WHP vision, strategies and action plans, and for reporting on progress. Grant funding was available to departments as an incentive for WHP including for example, development of a workplace health promotion resource toolkit, funding equipment or recreation spaces, development of a computer-based system to interrupt sitting time and prompt healthy activity, and individual assessment, activity or education programs. The number of departments with an established WHP program increased from 6 in 2009 to 13 in 2012. The mean number of initiatives per department increased from 13 in 2009 to 48 in 2012 (Additional file 1: Figure S1).

#### Exposures

Healthy@Work strategies targeted i) individuals via mental health and well-being, health education, health assessments, physical activity, and injury management, and ii) organizational change through initiatives such as increasing physical space for health-activities, making healthy food options available, funding onsite gymnasiums, giving access to stairs, promoting health via information bulletins and implementing health-promoting policies. Primary job stress prevention strategies (e.g., job control) were not included in Healthy@Work.

For analysis of individual exposures we first calculated a score indicating the ‘*availability’* of Healthy@Work strategies [41]. This score was obtained from questions asking respondents to provide a ‘yes’ or ‘no’ answer to a specified list of Healthy@Work amenities and programs (Additional file 2: Figure S2). The availability timeframe was *‘the previous 12 months’* in 2010 as a baseline reference period and *‘the previous 3 years’* in 2013 to capture the period over which Healthy@Work was implemented. A ‘total availability’ score was derived from per-person counts of positive responses to question items and a minimum score of 1 was needed to calculate participation. This approach was taken so that respondents could distinguish between work situations were WHP interventions were available (including health-promoting environments), and where they actually participated in activities. Where participants provided a ‘yes’ answer to activities, they were asked for the number of times they had participated and we used this information to calculate a total participation score per person (Additional file 2: Figure S2 describes calculations).

### Outcomes

Our overall measure of job stress outcome was Effort-reward imbalance (ERI). We applied the 17 item ERI questionnaire, which is a validated self-report survey with 6 items measuring Effort and 11 items dedicated to Reward [42]. A ratio is typically calculated for every person by first adding all scores for each of the effort (*e*) and reward (*r*) scales, then applying the formula *e/(r* x *c)* where *c* equals the proportion 6/11. Scores ≥1 are argued to indicate job strain conditions. The procedure for calculating the Reward component and its subscales of self-esteem, job security and career advancement has been described elsewhere [42]. Continuous scale scores were used to maximize the data available for analysis.

### Participants and sample size

We collected data via repeated, cross-sectional postal survey (2010 and 2013), selecting a 40 % random population sample from the total pool of workers, stratified according to employment condition, employment category and department (Additional file 3: Figure S3). In 2013 a portion of workers were re-selected by chance and survey respondents from this group were referred to as the ‘cohort’ (men = 161; women = 423). Survey responses were merged with de-identified administrative data and this process enabled propensity weighting to adjust for possible non-response bias (described below).

### Statistical analysis and methods

The repeated cross-sectional surveys were analyzed together in two-stages: 1) assessing whether mean ERI or its subcomponent scores changed over time and estimating associations between these scores and the availability of, or participation in Healthy@Work programs in 2010 and 2013; and 2) assessing whether there were changes in availability or participation over time. Survey responses were anticipated to be more similar within government departments, and for those who were in the cohort of repeat respondents. Mixed-effects linear regression modelling with random intercepts for department and participants was used to allow for correlated responses. Models were stratified by sex due to known differences in employee reporting of psychological distress. In stage 1, linear mixed-models were constructed with the outcome ERI (or its subcomponents) and a dummy variable for *‘survey year’* in the fixed effect section of each model along with covariates for confounders [43]. This process allowed us to determine whether ERI scores or their components changed by survey year. We then added covariates for total availability or participation. We tested for interaction between survey year and Healthy@Work exposure variables in each model to assess whether the effect of exposure changed between surveys. Confounders were identified via regression modelling techniques described by Hosmer, Lemeshow and Sturdivant [44] and were defined as those variables that were associated with the outcome and which also produced more than 10 % change in an estimated coefficient of the model.

Poisson regression with random effects as above was used to assess whether mean availability of, or participation in Healthy@Work strategies had changed over time. Model diagnostics from linear mixed effects models showed that residuals were skewed and an inverse transformation was applied to the ERI values. We then back-transformed the ERI results to present mean estimates on the original scale of measurement. Further we applied propensity weighting as described by Little and Rubin [45] to deal with potential non-response bias; the propensity model included age, sex, government department, employment category, employment condition, and tenure using the human resources administrative database as the reference population.

Models showing relationships between the exposure and outcome were corroborated by replicating the analysis with the repeat-respondent cohort. All analyses were conducted using STATA 12.1 (StataCorp LP, Texas, USA).

## Results

### Participants

Survey response proportions were 28 % (*n* = 3406) in 2010 and 27 % (*n* = 3228) in 2013. When compared with non-responders, responders tended to be older, have longer average tenure, and for women, be permanent employees (Additional file 4: Table S1). Weighting addressed these response variations. Table 1 shows basic respondent characteristics across both time-points. Men were proportionally more likely to be full-time employed (84 % in 2013) than women (48 % in 2013).

**Table 1:** Respondent characteristics for the 2010 and 2013 *Partnering* Healthy@Work workforce surveys

	Men					Women				
	2010		2013			2010		2013		
Continuous Variables	Mean	SD	Mean	SD	*p*	Mean	SD	Mean	SD	*p*
Age [years, mean] (SE)	47.1	10.1	47.6	10.4	0.212	45.8	10.4	46.8	10.3	0.001
Tenure (SE)	14.1	11.8	14.9	11.7	0.125	12.7	10.2	13.0	10.3	0.211
Hours worked (SE)	40.4	12.9	40.1	13.4	0.667	36.8	15.7	36.0	15.6	0.078
Annual Salary ($ AU)^a^	66,566	20,487	73,608	36,033	<0.001	63,232	20,073	70,091	20,333	<0.001
Categorical Variables	%	n	%	n		%	n	%	n	
Marital Status										
Married/ Partner	91	774	94	763	ref	85	1767	85	1711	ref
Not married	9	79	6	52	0.029	15	313	15	301	0.937
Education										
Post school	61	495	66	514	ref	68	1308	66	1239	ref
Middle school	3	23	2	12	0.053	2	40	2	36	0.826
Upper school	36	291	32	248	0.064	30	567	32	606	0.087
Employment band										
Low/mid band	75	719	78	716	ref	89	2183	94	2166	Ref
High/very high band	25	245	22	200	0.068	11	261	6	145	<0.001
Employment Category										
Employment Category	88	848	86	785	ref	92	2256	88	2034	ref
Fixed-term/ casual	12	116	14	131	0.146	8	188	12	276	<0.001
Employment Condition										
Full-time	84	814	84	772	ref	51	1243	48	1105	ref
Part-time	16	150	16	144	0.924	49	1201	52	1206	0.036
Days worked										
Mon to Fri	69	665	75	646	ref	53	1289	63	1205	ref
Days Vary Weekly	18	170	19	160	0.796	16	382	19	367	0.739
Other	13	122	6	55	<0.001	31	759	18	355	<0.001
Total Respondents		964		917			2444		2311	

^a^Based on full-time equivalent hours

The cohort group had some distinguishing features from the general respondent group (Additional file 5: Table S2) with men working longer hours, having higher average education, slightly more permanency of employment and higher average tenure overall. Women in the cohort also had higher tenure, higher average age and were more likely to be in permanent positions.

### Availability of and participation in workplace health promotion

**Fig. 1:**
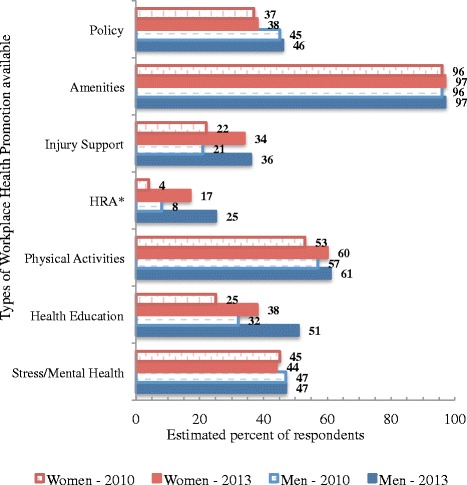
The range of available Healthy@Work initiatives in 2010 and 2013 reported by respondents in 2010 and 2013

Estimated percentages of workers reporting availability of different types of Healthy@Work initiatives in 2010 and 2013 are illustrated in Fig. 1. Poisson modelling (Additional file 6: Table S3) showed that WHP availability was 14 % higher (for men and women) in 2013 (95 % CI: 12 % to 17 %). The number of times men and women participated across all programs had approximately doubled in 2013.

### Univariable correlates of effort reward imbalance

Covariates univariably associated with ERI were age, marital status, annual salary, education, employment band, employment category, employment condition, tenure, hours worked and regular work-days (Table 2). In subsequent model testing, age was a confounder for availability and age and employment band were confounders for participation among men. However the models presented here were only adjusted for age so as to standardize comparisons across all models and maximize discussion across the whole employee group. Had the models all been adjusted for employment condition, description would have been narrowed to reference the higher-band employee group.

**Table 2:** Univariable associations between Effort-Reward Imbalance and respondent characteristics stratified by sex and survey year

	Men						Women					
	2013			2010			2013			2010		
Effort-Reward Imbalance	ß	95 % CI		ß	95 % CI		ß	95 % CI		ß	95 % CI	
Age (continuous)	0.001	−0.001	0.001	−0.001	−0.002	0.000	0.000	−0.001	0.001	0.001	0.000	0.001
Marital status												
Married/Partner	ref			ref			ref			ref		
Not married	0.013	−0.047	0.072	−0.026	−0.051	−0.051	0.012	−0.007	0.032	0.013	−0.007	0.033
Annual salary	0.011	−0.003	0.003	0.002	−0.005	0.010	0.010	0.002	0.019	0.011	0.000	0.022
Education												
Post school	ref			ref			ref			ref		
School	−0.023	−0.020	0.032	−0.020	−0.040	0.000	−0.030	−0.046	−0.014	−0.023	−0.038	−0.008
Band												
Low/mid	ref			ref			ref			ref		
High/very high	0.039	−0.023	0.024	0.024	0.003	0.003	0.020	−0.003	0.043	0.039	0.019	0.059
Employment category												
Permanent	ref			ref			ref			ref		
Fixed term/casual	−0.026	−0.089	−0.033	−0.028	−0.056	−0.001	−0.034	−0.053	−0.015	−0.026	−0.048	−0.003
Employment condition												
Full-time	ref			ref			ref			ref		
Part-time	−0.029	−0.058	−0.006	−0.030	−0.055	−0.005	−0.035	−0.048	−0.022	−0.029	−0.041	−0.017
Tenure	0.002	−0.001	0.001	0.001	0.000	0.002	0.001	0.001	0.002	0.002	0.002	0.003
Regular day worked												
Yes	ref			ref			ref			ref		
No	−0.032	−0.058	−0.006	−0.028	−0.049	−0.008	−0.040	−0.058	−0.022	−0.032	−0.048	−0.017
Hours worked (continuous)	0.002	0.001	0.003	0.003	0.002	0.003	0.002	0.002	0.002	0.002	0.002	0.002

### Repeated cross-sectional modelling

#### Changes in effort reward imbalance over time

Table 3 shows that men’s ERI score estimates excluding exposure to WHP were approximately 4 points higher over time (*p* < 0.001), with corresponding increases in perceived effort and decreases in perceived reward, including its subcomponents of self-esteem, job security and career promotion. These results indicate there were basic increases in ERI scores for men in 2013 (i.e., time-based differences) that were not accounted for by the socio-demographic factors or work characteristics measured here. Women’s results indicate mean ERI scores were less over time, but were not statistically different between 2010 and 2013 (*p* =0.414).

**Table 3:** Linear mixed models for all respondents regressing Effort Reward Imbalance on Workplace Health Promotion^e^ exposures

	Men							Women						
	2010			2013				2010			2013			
Outcome measure	β^a^	95 % CI		β	95 % CI		*p* ^b^	β^a^	95 % CI		β	95 % CI		*p*
ERI (mean)	0.371	0.351	0.351	0.410	0.384	0.435	<0.001	0.373	0.357	0.357	0.366	0.344	0.387	0.414
Reward	47.88	45.63	50.13	44.97	42.28	42.28	<0.001	47.41	46.71	48.10	47.73	47.19	48.26	0.267
Self-esteem	21.95	21.26	22.64	21.10	20.29	21.90	0.002	21.65	21.20	22.10	21.65	21.32	21.98	0.897
Job security	8.77	7.84	9.70	7.12	6.09	8.14	<0.001	8.39	8.22	8.55	8.26	7.88	8.63	0.488
Career promotion	16.32	14.96	17.69	15.10	13.66	16.54	<0.001	16.36	15.96	16.76	16.68	16.34	17.01	0.010
Effort	10.13	9.69	10.56	10.80	10.40	11.21	<0.001	9.99	9.60	10.38	9.85	9.31	10.38	0.494
Exposure	β^c^	95 % CI		β	95 % CI		*p*	β^c^	95 % CI		β	95 % CI		*p*
*WHP Availability*	(~n = 947)			(~n = 906)				(~n = 2396)			(~n = 2283)			
ERI	−0.006	−0.011	−0.001	−0.008	−0.013	−0.002	0.008	−0.003	−0.001	0.003	−0.004	−0.007	−0.002	0.002^d^
Reward	0.332	−0.185	0.848	0.295	−0.156	−0.156	0.218	0.161	0.029	0.293	0.154	0.020	0.288	0.020
Self-esteem	0.040	−0.317	0.397	0.037	−0.295	0.369	0.827	0.135	0.062	0.207	0.128	0.050	0.206	<0.001
Job security	0.059	−0.018	0.136	0.148	0.012	0.283	0.043^d^	−0.042	−0.096	0.012	−0.035	−0.090	0.019	0.135
Career promotion	0.088	−0.034	0.211	0.076	−0.026	0.178	0.167	0.027	−0.080	0.134	0.026	−0.087	−0.087	0.627
*WHP Participation*	(~n = 735)			(~n = 759)				(~n = 1804)			(~n = 1881)			
Effort	−0.024	−0.036	−0.012	−0.023	−0.035	−0.012	<0.001	0.011	−0.015	0.036	0.011	−0.013	0.034	0.420

^a^Estimated scale score excluding exposure after back-transformation and controlling for confounders

^b^ *p*-value of linear mixed models regressing the outcome measure on survey year. Models were adjusted for age

^c^Values represent the results from linear mixed models including exposure variable. Beta values have been back-transformed to estimate the coefficient on the original scale

^d^Represents interaction term present in model

^e^The results are based on a composite measure that includes all forms of workplace health promotion [WHP] (i.e., policy, amenities, injury support, health risk assessment, physical activities, health education, stress/mental health)

#### Changes in perceived effort and reward in association with WHP

At baseline, Table 3 also shows an inverse and additive relationship was identified between higher participation in WHP and lower effort scores for men [ß = −0.024, 95 % CI: −0.036 to −0.012]. Over time the magnitude of effect for this association increased (*p* = <0.001) but estimated beta-values were modest overall. Sub-analysis of this model (not shown) adjusting for employment band and age was slightly more conservative [ß = −0.025, 95%CI: −0.043 to −0.007 (*p* = 0.009)]. Statistical associations between WHP availability and reward (and its subcomponent self-esteem) were neither present at baseline nor over time for men (*p* = 0.218). Similar results were also found for the reward sub-component, self-esteem (*p* = 0.827) for this group.

For women, no statistical relationship was identified between WHP participation and effort at either time point (*p* = 0.420). However an additive association was found at baseline between higher WHP availability and higher perceived reward [ß = 0.161, 95 % CI: 0.029 to 0.293] that included higher self-esteem for this group. Over time the magnitudes of effect for these associations both increased (reward: *p* = 0.020; self-esteem: *p* < 0.001) among women but did not translate to a statistical difference in either reward or self-esteem in 2013.

### Cohort analyses

To corroborate the effects observed in repeated cross-sectional analyses we replicated our models using confirmatory evidence from the cohort of repeat responders (Table 4). The model results show a high degree of overlap for coefficient estimates of the cohort and general respondent populations. However, for the model regressing job security on WHP availability, the strength of association changed over time (*p* = 0.065) for the cohort group but not within the whole respondent population (*p* = 0.135).

**Table 4:** Linear mixed models for the cohort group regressing Effort Reward Imbalance on Workplace Health Promotion^e^ exposures

	Men (n = 161)							Women (n = 423)						
	2010			2013				2010			2013			
Outcome measure	ß^a^	95 % CI		ß	95 % CI		*p* ^b^	β	95 % CI		β	95 % CI		*p* ^b^
ERI (mean)	0.379	0.353	0.404	0.422	0.389	0.457	<0.001	0.376	0.342	0.411	0.369	0.330	0.408	0.496
Reward	49.47	47.98	50.96	46.42	46.42	48.58	0.012	45.62	40.27	50.96	45.93	40.61	51.26	0.237
Self-esteem	22.54	21.98	23.07	21.73	20.99	22.48	0.010	20.78	17.90	23.67	20.80	18.06	23.53	0.961
Job security	9.26	8.05	10.48	7.12	6.16	8.07	<0.001	7.67	6.21	9.13	7.61	5.98	9.24	0.851
Career promotion	17.50	16.69	16.69	16.26	15.15	17.37	0.004	16.47	15.34	15.34	16.81	15.95	17.68	0.012
Effort	10.42	9.74	11.11	11.18	10.47	11.90	0.020	0.020	9.46	11.00	10.09	9.21	10.96	0.577
Exposure	β^c^	95 % CI		β	95 % CI		*p*	β	95 % CI		β	95 % CI		*p*
*WHP Availability*														
ERI	−0.006	−0.012	−0.001	−0.008	−0.014	−0.001	0.012	0.001	−0.001	0.003	−0.004	−0.007	−0.001	0.002^d^
Reward	0.338	−0.235	0.911	0.301	−0.201	0.802	0.251	0.124	0.006	0.242	0.118	0.004	0.239	0.072
Self-esteem	0.041	−0.339	0.421	0.038	−0.318	0.395	0.395	0.102	0.024	0.180	0.128	0.050	0.206	0.022
Job security	0.055	−0.042	0.151	0.131	−0.006	0.278	0.091^d^	−0.044	−0.092	0.006	−0.033	−0.081	0.014	0.065
Career promotion	0.104	−0.043	0.252	0.091	−0.033	0.215	0.173	0.023	−0.086	0.132	0.024	−0.092	0.140	0.684
*WHP Participation*														
Effort	−0.037	−0.064	−0.011	−0.036	−0.062	−0.011	0.003	0.014	−0.016	0.043	0.015	−0.016	0.046	0.117

^a^Estimated scale score excluding exposure after back-transformation and controlling for confounders

^b^ *p*-value of linear mixed models regressing the outcome measure on survey year. Models were adjusted for age

^c^Values represent the results from linear mixed models including exposure variable. Beta values have been back-transformed to estimate the coefficient on the original scale

^d^Represents interaction term present in model

^e^The results are based on a composite measure that includes all forms of workplace health promotion [WHP] (i.e., policy, amenities, injury support, health risk assessment, physical activities, health education, stress/mental health)

## Discussion

Our first hypothesis, that higher availability of WHP would be positively associated with perceived reward through improved self-esteem was supported among women but not men. Our second hypothesis, that higher participation in WHP would be negatively associated with perceived effort was supported for men but not women. However, the magnitudes of effect for these additive associations were modest and were not reflected as statistical differences in perceived effort or reward (including self-esteem) at a working-population level over time. We found a high corroboration between results for the repeat-responder cohort and the broader respondent group, which was randomly sampled and weighted to minimize non-response bias. Therefore these results seem generalizable to the source population of public sector workers under study.

To show effects at a population level, additive relationships rely on increased dosage of exposure (e.g., higher volumes of availability or higher participation levels). In 2013 self-reported WHP availability increased by 14 % and participation approximately doubled over time. Systematic differences in occupational exposures between sexes, linked to disparities in perceptions and/or reporting and variations in exposure between or within jobs [46] may also have contributed to our results. Recently published results from this project have indicated that where activities were available, participation was less likely among employees with cardio-metabolic conditions, those who smoked and workers with variable work schedules. Participation was more likely among administrative staff and those who participated in leisure time physical activity [47].

For women, we infer WHP availability contributed to perceptions of organisational support thereby enhancing self-esteem [20]. The ERI self-esteem construct was derived from items capturing perceptions of i) respect from supervisors and colleagues, ii) adequacy of support in difficult situations, and iii) effects of job interruptions [42]. Research using this concept of social exchange for other forms of non-monetary employee benefits, such as manager trustworthiness and procedural justice has supported their relationship with job satisfaction and employee turnover [48]. However, the increases in WHP availability may have been of insufficient dose, or may have needed supplement from other non-monetary benefits to show changes in self-esteem at a population-level. A study of Chinese physicians, by Li and colleagues [49] has found differences in the way men and women perceive the reward sub-factor of ERI when facing similar work environments.

The time period for Healthy@Work implementation coincided with the global economic downturn, which had major financial ramifications for the Tasmanian Government. During the implementation period, government directives also focused on long-term reduction of operating costs, including labor costs via vacancy control and productivity management. For men, who were higher wage-earners and more likely to be working in full-time or management positions, it is possible that the adverse events reported here may have contributed to perceived or real threats of job-loss and work intensification at population-level [50]. Higher WHP participation may have enhanced work-related coping or personal well-being but it was only one side of the effort-reward equation. We interpret that men did not perceive WHP availability as a reward. It is possible that men in this workforce were more sensitive to job security than socio-emotional relationship issues [51]. We note that sex-based differences in occupational exposures been noted previously [46] and the distinctions identified by employment band for participating men require further investigation. However, attention to areas such as self-esteem, job security and promotion prospects through stress management programs or primary stress prevention interventions may have been more suited to addressing increased job stress among men.

### Limitations

Repeated-cross sectional designs offer advantages in cost and allow for changes in working population characteristics but they do not allow causal inferences. Neither do they control for baseline differences in exposure to interventions or between individuals, or influences on results due to inter-departmental migration, [52, 53]. Other research has shown that for large population samples repeated cross-sectional designs can be superior to cohort designs [53]. Linear mixed-modelling analysis also provides robust estimates in the face of modest associations [54]. Further, even though response rates were arguably low, they were typical for organizational surveys [55] and have been addressed here through weighting procedures. We acknowledge it is possible that people with greater stress may have chosen not to respond to the surveys [56, 57]. Our study did not measure societal trends and commonly changing features of public sector workforces may have influenced the observed changes in effort and reward over time. Furthermore, our self-reported measures of exposure may have been too crude. The focus of Healthy@Work was on comprehensive intervention and departmental programs were necessarily different, catering for working circumstances, and employee needs and preferences. This meant that activities also had different levels of content, intensity and levels of delivery and were implemented across a large and diverse working population. Furthermore, participation questions did not ask about the specific dose of activity (i.e., whether the number of times represented a full dose or its portion). Other authors have acknowledged the innate challenges in measuring customized WHP programs [58], and in this setting although the study’s design did not capture specific details of availability or participation, it was able to deal with the heterogeneity of comprehensive WHP. We do not know whether the changes in wording of the response period for our exposures affected the results but this approach enabled us to capture the full period of WHP. The self-reported increases in WHP availability appeared to reflect increases in departmental data obtained from the employers’ audit processes (Additional file 1: Figure S1) but further investigation is needed to assess self-reported versus actual overlap. More detail on specific types of interventions from organizations would have been an advantage. Identification of further exposure effects may require differently timed data collection. Recall bias can also be an issue in self-reported data [59]. Broader conclusions about the generalizability of this study would benefit from follow-up research in other public or private sector workplaces.

## Conclusions

This research provides much-needed evidence of potential benefits obtained from a comprehensive WHP intervention in a naturalistic setting. Interesting gender differences were observed with WHP availability associated with a sense of reward via enhanced self-esteem among women, and WHP participation associated with lower perceived effort in men. Gains associated with comprehensive WHP were modest over time and men in particular may have benefitted from more traditional preventative stress management interventions. These findings appeared generalizable to the general population of public sector workers.

### Availability of data and materials

Data supporting these findings are held by the Menzies Institute for Medical Research and requests for information should be directed to the corresponding author.

## Additional files

Additional file 1: Figure S1. Mean numbers of available Workplace Health Promotion (WHP) initiatives per department reported through Tasmanian Government audits between 2009 and 2012. (PDF 77 kb) Additional file 2: Figure S2. Question items used in the 2013 Partnering Healthy@Work survey to calculate workplace health promotion exposures for availability and participation. (PDF 171 kb) Additional file 3: Figure S3. Flowchart showing sampling and responses to the Partnering Healthy@Work surveys as at November 2014. (PDF 94 kb) Additional file 4: Table S1. Comparisons of respondents and non-respondents for Partnering Healthy@Work surveys in 2010 and 2013 by age, tenure, employment condition and employment category, stratified by sex. (PDF 56 kb) Additional file 5: Table S2. Cohort characteristics for the 2010 and 2013 Partnering Healthy@Work surveys. (PDF 59 kb) Additional file 6: Table S3. Ratios of mean reported availability of and participation in Healthy@Work initiatives in 2013 relative to 2010. (PDF 69 kb)

## Abbreviations

WHP: Workplace Health Promotion
ERI: Effort Reward Imbalance
CI: Confidence Interval
