# Supplementary material for: Does workplace health promotion contribute to job stress reduction? Three-year findings from Partnering Healthy@Work
Source: BMC Public Health. 2015 Dec 24;15:1293. doi: 10.1186/s12889-015-2625-1 (PMC4690240; doi:10.1186/s12889-015-2625-1)
Supplement: Additional file 1: Figure S1. — Mean numbers of available Workplace Health Promotion (WHP) initiatives per department reported through Tasmanian Government audits between 2009 and 2012. (PDF 77 kb) [file 12889_2015_2625_MOESM1_ESM.pdf]

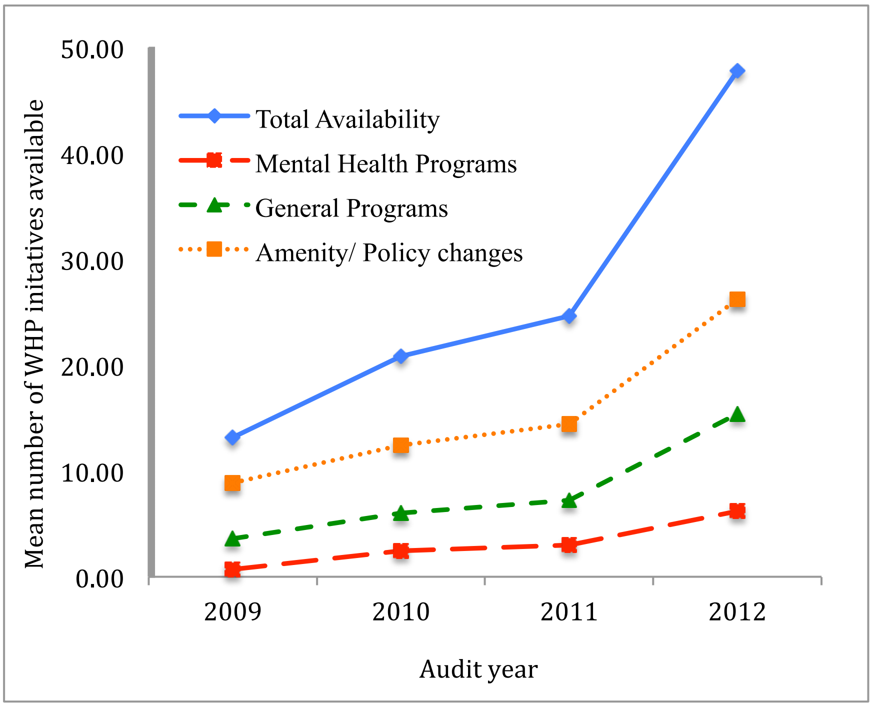

Additional Figure 1. Mean numbers of available Workplace Health Promotion (WHP) initiatives per department reported through Tasmanian Government audits between 2009 and 2012.
