# Supplementary material for: Does workplace health promotion contribute to job stress reduction? Three-year findings from Partnering Healthy@Work
Source: BMC Public Health. 2015 Dec 24;15:1293. doi: 10.1186/s12889-015-2625-1 (PMC4690240; doi:10.1186/s12889-015-2625-1)
Supplement: Additional file 2: Figure S2. — Question items used in the 2013 Partnering Healthy@Work survey to calculate workplace health promotion exposures for availability and participation. (PDF 171 kb) [file 12889_2015_2625_MOESM2_ESM.pdf]

Additional Figure 2. Question items used in the 2013 Partnering Healthy@Work survey to calculate workplace health promotion exposures for availability and participation.

The following items are about health activities in your workplace for your main job in the Tasmanian State Service.

32. Please indicate the amenities/supports that are available. Choose all that apply.

- O Space to hold activities
- O Equipment storage areas
- O Lunch / break room
- O Onsite gymnasium / fitness centre
- O Bicycle racks/storage
- O Healthy vending machine options
- O Workplace Wellness Health Champions
- O Shower and change facilities
- O Fruit baskets provided
- O Outdoor exercise areas for employees to use
- O Stairs / stair wells that can be used for exercise
- O Healthy food options (e.g work meetings, on-site)
- O Drinking water
- O Flexible work arrangements
- O Bulletin boards, newsletters, emails or websites where health information is provided

O Other (please specify) ____

33. Please indicate which workplace health and wellbeing activities were available in the past 3 years. If 'yes', please indicate the number of times you participated.

	Available in the past 3 years		Number of times you participated in the past 3 years
**Type of health and wellbeing activities available**	**No**	**Yes**	**If yes, please enter a number**
**a) Education**<br>e.g. Health information seminars or workshops	○	○	☐☐☐
**b) Health assessments**<br>e.g. Health checks (BUPA/MBF health lounges), regular health assessments, regular fitness assessments, pre-employment health screening	○	○	☐☐☐
**c) Physical activity**<br>e.g. Global Corporate Challenge, Hydra-Walk, organisation sport team, sport or activity days, exercise or physical activity sessions (e.g. yoga, fit-ball, boot camp), active transport (e.g. TravelSmart Workplace Program, cycle to work or walk to work)	○	○	☐☐☐
**d) Smoking**<br>e.g Smoking cessation programs (e.g. nicotine replacement, counselling)	○	○	☐☐☐
**e) Mental health and wellbeing**<br>e.g. Employee Assistance Programs, stress-management program or strategies, allocated stretching or relaxing times, massage, personal development opportunities for life skills, training or activities for mental health and wellbeing (e.g. Mental Health First Aid, Mindfulness, Flourishing People Happiness Training, *beyondblue*)	○	○	☐☐☐
**f) Interrupted sitting**<br>e.g. Exertime, Project Pause, standing work station	○	○	☐☐☐
**g) 'Walk and talk' or active meetings**	○	○	☐☐☐
**h) Flu vaccination**	○	○	☐☐☐
**i) Injury prevention/rehabilitation**	○	○	☐☐☐
**j) Subsidised membership to off-site facilities or programs**	○	○	☐☐☐
**k) Regular health and wellbeing activities facilitated by the organization**<br>e.g. walking/cycling groups	○	○	☐☐☐
**l) Other**<br>(Please specify)____________________________	○	○	☐☐☐

If you did not participate in any of the above activities, skip to question 35.

Availability is calculated as a total count by adding each indicated item in question 32 (a mark = 1) to each item marked 'yes' (yes = 1) in question 33 under the section header 'available in the past…'. Sub categories under question 32 for bicycle racks/ storage facilities, drinking water and workplace wellness health champions were excluded from analysis as they were not present in the 2010 Healthy@Work survey. Question 33d was similarly excluded. Question 33h was also excluded as preliminary data analyses indicated it artificially skewed the responses. The response category wording was changed for question 33 from that for 2010 survey, which was worded

as ‘past 12 months’ rather than ‘past 3 years’ listed above, so as to capture the full period of intervention.

Participation is calculated from question 33 from answers given under the heading ‘number of times you participated…’ and only for instances where the availability score is greater than zero. Question 33 sub-items excluded from the availability analysis are also excluded from the participation score. The remaining items are added to provide a total score for participation per respondent.
