# Supplementary material for: Does workplace health promotion contribute to job stress reduction? Three-year findings from Partnering Healthy@Work
Source: BMC Public Health. 2015 Dec 24;15:1293. doi: 10.1186/s12889-015-2625-1 (PMC4690240; doi:10.1186/s12889-015-2625-1)
Supplement: Additional file 3: Figure S3. — Flowchart showing sampling and responses to the Partnering Healthy@Work surveys as at November 2014. (PDF 94 kb) [file 12889_2015_2625_MOESM3_ESM.pdf]

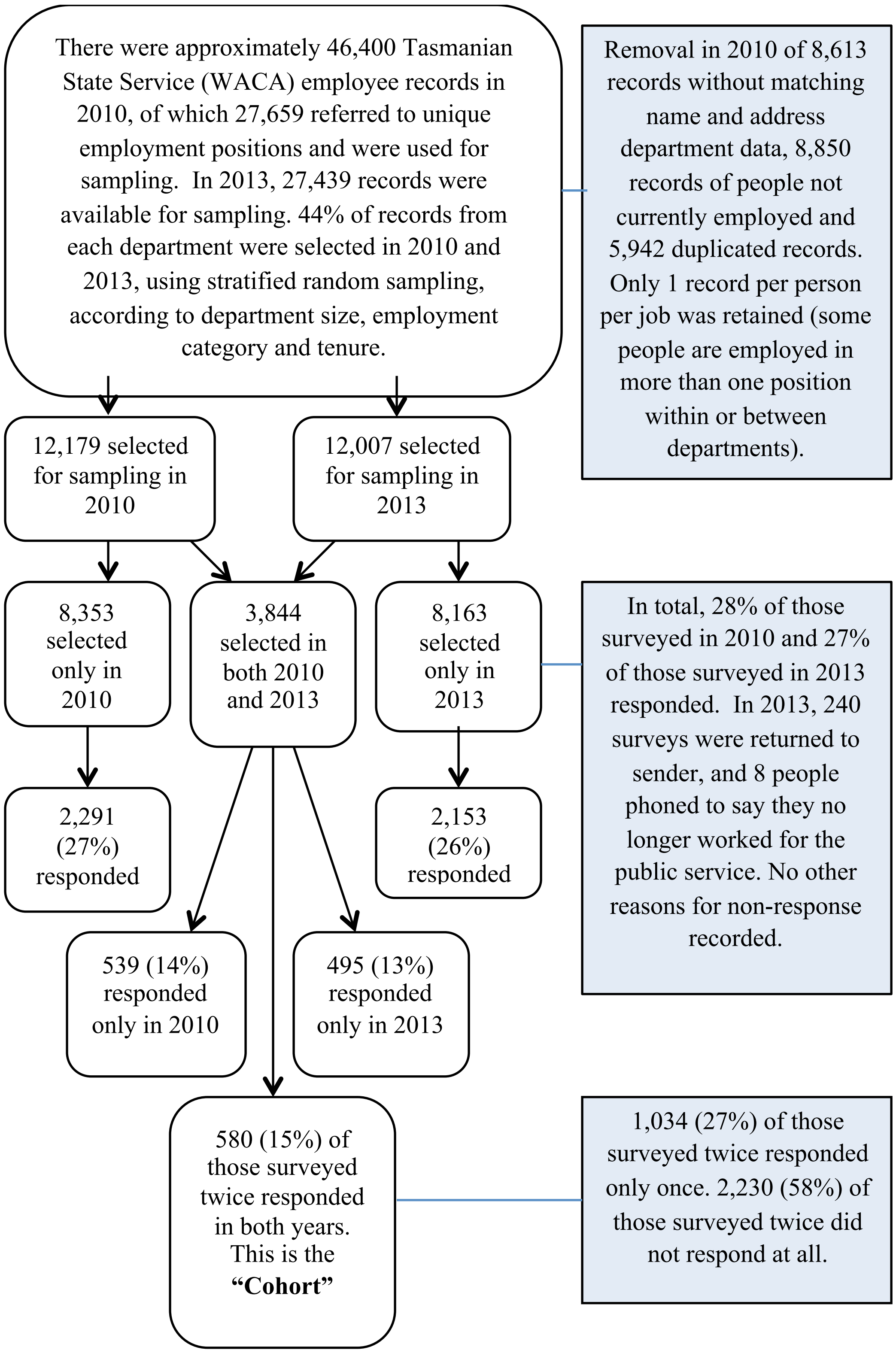

Additional Figure 3. Flowchart showing sampling and responses to the Partnering Healthy@Work surveys as at November 2014.
