# Supplementary material for: Does workplace health promotion contribute to job stress reduction? Three-year findings from Partnering Healthy@Work
Source: BMC Public Health. 2015 Dec 24;15:1293. doi: 10.1186/s12889-015-2625-1 (PMC4690240; doi:10.1186/s12889-015-2625-1)
Supplement: Additional file 4: Table S1. — Comparisons of respondents and non-respondents for Partnering Healthy@Work surveys in 2010 and 2013 by age, tenure, employment condition and employment category, stratified by sex. (PDF 56 kb) [file 12889_2015_2625_MOESM4_ESM.pdf]

Additional Table 1. Comparisons of respondents and non-respondents for Partnering Healthy@Work surveys in 2010 and 2013 by age, tenure, employment condition and employment category, stratified by sex

	**Respondents**	**Non-respondents**	**Test statistic (df)**	**p-value**
**2010**		**Men (n=4053)**		
n	947	3106		
Mean age (SD)	46.99 (0.34)	44.37 (0.19)	t(4051)=-6.6	<0.001
Mean tenure (years (SD))	14.18 (0.38)	12.37 (0.20)	t(4051)=-4.4	<0.001
% full-time	84.69	83.77	chi2(1)=0.5	0.500
% permanent	88.38	85.64	chi2(1)=4.6	0.030
**2013**		**Men (n=3865)**		
n	917	2948		
Mean age (SD)	47.64 (0.34)	44.87 (0.21)	t(3863)=-6.7	<0.001
Mean tenure (years (SD))	14.90 (0.39)	12.25 (0.20)	t(3863)=-6.5	<0.001
% full-time	84.30	79.85	chi2(1)=8.9	0.003
% permanent	85.71	84.46	chi2(1)=0.8	0.360
**2010**		**Women (n=8126)**		
n	2422	5704		
Mean age (SD)	45.84 (0.21)	44.36 (0.14)	t(8124)=-5.7	<0.001
Mean tenure (years (SD))	12.72 (0.21)	11.41 (0.13)	t(8124)=-5.4	<0.001
% full-time	50.87	48.28	chi2(1)=4.5	0.030
% permanent	92.53	89.46	chi2(1)=18.3	<0.001
**2013**		**Women (n=8142)**		
n	2311	5831		
Mean age (SD)	46.80 (0.21)	44.80 (0.15)	t(8140)=-7.3	<0.001
Mean service length (years (SD))	13.03 (0.21)	11.98 (0.13)	t(8140)=-4.2	<0.001
% full-time	47.81	43.32	chi2(1)=13.5	<0.001
% permanent	88.01	85.10	chi2(1)=11.6	0.001
