# Supplementary material for: Does workplace health promotion contribute to job stress reduction? Three-year findings from Partnering Healthy@Work
Source: BMC Public Health. 2015 Dec 24;15:1293. doi: 10.1186/s12889-015-2625-1 (PMC4690240; doi:10.1186/s12889-015-2625-1)
Supplement: Additional file 5: Table S2. — Cohort characteristics for the 2010 and 2013 Partnering Healthy@Work surveys. (PDF 59 kb) [file 12889_2015_2625_MOESM5_ESM.pdf]

Additional Table 2 Cohort characteristics for the 2010 and 2013 *Partnering* Healthy@Work surveys.

	Men (n = 161)					Women (n= 423)				
	2010		2013			2010		2013		
	Mean	SD	Mean	SD	p^2	Mean	SD	Mean	SD	p
Continuous Variables										
Age [years, mean] (SE[1]	44.1	0.75	46.9	0.79	0.210	45.8	0.46	48.8	0.47	<0.001
Tenure (SE)	13.3	0.81	16.5	0.84	0.032	12.66	0.47	15.7	0.48	<0.001
Hours worked (SE)	43.0	1.10	41.17	1.01	0.007	36.9	0.78	36.7	0.80	0.146
Annual Salary ($AU)1	74,182	2797	84554	2746	0.101	61,865	861	71,687	908	0.354
Categorical Variables	%	n	%	n		%	n	%	n	
Marital Status										
Married/ Partner	94	133	93	138	ref	83	309	85	324	ref
Not married	6	9	7	10	0.365	17	62	15	59	0.375
Education										
Post school	66	88	68	92	ref	67	211	65	219	ref
Middle school	-	-	-	-	0.006	2	4	2	7	0.583
Upper school	34	46	32	44	0.569	32	100	33	109	0.482
Employment band										
Low/mid band	74	119	77	124	ref	90	379	91	386	ref
High/very high band	26	42	23	37	0.689	10	44	9	37	0.234
Employment Category										
Permanent	90	145	90	146	ref	95	403	98	414	ref
Fixed-term/ casual	10	16	10	15	0.041	5	20	2	9	<0.001
Employment Condition										
Full-time	90	146	88	142	ref	53	226	49	207	ref
Part-time	10	15	12	19	0.006	47	197	51	216	0.248
Days worked										
Mon to Fri	76	122	75	118	ref	54	229	66	221	ref
Days Vary Weekly	18	28	18	29	0.659	14	59	17	59	0.148
Other	6	11	7	11	0.054	32	133	17	59	0.555

[1] Based on full-time equivalent hours.

[2] Comparsion made between total cohort group and total survey respondent group.
