# Supplementary material for: Does workplace health promotion contribute to job stress reduction? Three-year findings from Partnering Healthy@Work
Source: BMC Public Health. 2015 Dec 24;15:1293. doi: 10.1186/s12889-015-2625-1 (PMC4690240; doi:10.1186/s12889-015-2625-1)
Supplement: Additional file 6: Table S3. — Ratios of mean reported availability of and participation in Healthy@Work initiatives in 2013 relative to 2010. (PDF 69 kb) [file 12889_2015_2625_MOESM6_ESM.pdf]

Additional Table 3 Ratios of mean reported availability of and participation in Healthy@Work initiatives in 2013 relative to 2010.

	Men						Women					
	**Mean (SE)**		**Mean Ratio**[1]	**95% CI**		***p***	**Mean (SE)**		**Mean Ratio**	**95% CI**		***p***
**Availability**	*2010*	*2013*					*2010*	*2013*				
Total	0.41 (0.01)	0.46 (0.01)	1.14	1.10	1.19	<0.001	0.38 (<0.01)	0.42 (0.01)	1.14	1.11	1.17	<0.001
**Participation**												
Total	1.97 (0.24)	4.85 (1.20)	1.93	1.71	2.17	<0.001	1.66 (0.11)	3.77 (0.29)	2.16	2.00	2.34	<0.001

[1] Ratio of estimated mean exposures to Healthy@Work in 2013 relative to 2010.
